# Ignoring regression to the mean leads to unsupported conclusion about obesity

**DOI:** 10.1186/s12966-015-0212-6

**Published:** 2015-05-07

**Authors:** Asheley Cockrell Skinner, Steven B Heymsfield, Angelo Pietrobelli, Myles S Faith, David B Allison

**Affiliations:** The University of North Carolina at Chapel Hill, Chapel Hill, USA; Pennington Biomedical Research Center, Baton Rouge, USA; Verona University Medical School, Verona, Italy; Department of Biostatistics, University of Alabama at Birmingham, Birmingham, USA; Nutrition Obesity Research Center, University of Alabama at Birmingham, Birmingham, USA; Office of Energetics, University of Alabama at Birmingham, Birmingham, USA

## Abstract

Childhood obesity remains a substantial health concern for our population and thoughtful attempts to develop and evaluate the utility of programs to reduce childhood obesity levels are needed. Unfortunately, we believe the conclusion by Burke et al. that the HealthMPowers program produces positive change in body composition is incorrect because the results obtained are likely due to regression to the mean (RTM), a well-known threat to the validity of studies that is often overlooked. Using empirical data, we demonstrate that RTM is likely to be the cause for the changes reported. A more reasonable conclusion than the one of effectiveness the authors offered would be that the results did *not* support the effectiveness of the intervention. Public health officials, parents, school leaders, community leaders, and regulators need and deserve valid evidence free from spin on which they can base decisions.

Childhood obesity remains a substantial health concern for our population and thoughtful attempts to develop and evaluate the utility of programs to reduce childhood obesity levels are needed. For such efforts to be valuable, a sound evidential base is needed. As Casazza and Allison [1] wrote, we need probative research in this domain, that is, research which actually answers questions and meaningfully moves knowledge in the field forward. Given this, we read with interest the paper by Burke et al. [2] which implemented “A holistic school-based intervention for improving… body composition… in elementary school students” and concluded “The present report demonstrates the effectiveness of the HealthMPowers program in producing positive change in… student body composition…” Unfortunately, we believe that this conclusion is unreasonable because the results obtained do not demonstrate effectiveness.

## Threats to validity of inference

Regarding statements of causal inference, single group research designs have well-established threats to the internal validity summarized in the classic works of Cook and Campbell [3]. Two such threats are particularly pertinent to the present study and the conclusions that it can meaningfully support: (1) “history,” or historical events (either global or local) that occur at the same time as an intervention which might in actuality have influenced the presumed intervention effect; and (2) “regression to the mean” (RTM), a statistical phenomenon in which scores on average show improvements upward or downward towards the mean over time. Burke et al. do mention the potential effect of history in their limitations. However, RTM is an equally important threat to internal validity and the one we address herein.

## Regression to the mean as a particular threat to causal inference

First formally recognized by Galton over a century ago [4], regression to the mean (RTM) is sometimes seen as a function of measurement error. Although measurement error can produce RTM, RTM can occur in the absence of measurement error [5]. Although RTM applies to other bivariate distributions as well [6], RTM is most easily conceptualized in bivariate normal distributions. Whenever data are collected on two bivariate normal variables on a set of cases (e.g., children in a study), RTM will occur if the two variables are not perfectly correlated. This is true regardless of whether there is measurement error, regardless of the order of measurement, and regardless of whether the two variables are repeated measures of the same construct. One aspect of RTM especially critical for obesity intervention evaluation is that if a set of cases is selected for their deviations from the mean on one variable (e.g., z-BMI at time 1), they will, on average, be closer (in standard deviation units) to (i.e., ‘regress to’) the mean on another variable (e.g., z-BMI at time 2).

Unfortunately, RTM is often neglected in reasoning about effects [7]. For example, neglect of RTM has erroneously led some authors to conclude that drugs which cause weight gain cause less weight gain among those with unusually high BMI levels [8]. It has led some authors to conduct a power analyses that does not make sense by assuming that without treatment, most BMI z-scores would not go down over time among a sample of children initially selected for high BMI z-scores [9]. It seems now that neglect of RTM has again reared its head.

## Empirical evaluation of the consistency of the reported results with RTM

Understanding the potential impact of RTM in this situation requires evidence of the expected changes among children not involved in an intervention (i.e., the changes that might have been seen in a control group). We do not have data for children in similar schools across time, but other longitudinal surveys can provide some evidence of what we would expect.

We used the National Longitudinal Survey of Youth, 1997 cohort (see: http://www.bls.gov/nls/nlsy97.htm). This longitudinal survey examines a nationally-representative group of children at two year intervals. We included children who had measured values of height and weight at both age 9 and age 11 (approximating the fourth grade), and examined the change in BMI z-score. For these children, the mean change in BMI z-score for girls was 0.21 for healthy weight, −0.12 for overweight, and −0.22 for girls with obesity; for boys the change was 0.15 for healthy weight, −0.10 for overweight, and −0.21 for boys with obesity. This empirical evidence raises doubt regarding all studies showing declines in z-scores among children with obesity that do not include an appropriate control group.

Burke et al. showed a school-year decline for girls with obesity of −0.10 (i.e., a reduction of 0.10 units), while we would expect a decline of −0.22 over two years with no intervention. Similarly, they show a decline of −0.12 for boys, while we expect a decline of −0.21. Given the known changes among children with obesity, it is not only plausible, but in fact probable, that the changes seen by Burke were simple RTM effects.

## Conclusions and recommendation

Before Burke et al. conducted their research, it was plausible that the particular intervention used involving promotion of “healthy eating and physical activity in schools” might reduce obesity levels in participating children. However, after this research finding of no significant reduction in BMI z-scores in the total sample and no greater reduction in BMI z-scores among the higher BMI children than one would expect by RTM, a more reasonable conclusion would have been “Our results did not support the effectiveness of our intervention and, though it remains possible that it is effective, perhaps alternative approaches should be tried.” Burke et al. [2] state “The HealthMPowers initiative was never designed to be a research study.” This presumably explains why there was no control group used, but it does not justify then drawing conclusions about purported demonstrations of effectiveness. Sadly, high-quality, well-designed research of interventions and policies throughout public health appears to be the exception, not the rule [10]. We recognize the difficulties in performing school-based research, but we must consider the conclusions that can be drawn from uncontrolled studies. Research on childhood obesity interventions and policies is desperately needed, but it must also provide real evidence of which policies are effective and for which groups of children [11]. Public health officials, parents, school leaders, community leaders, and regulators need and deserve valid evidence free from spin [12] on which they can base decisions.
